# Sex Differences in Domain‐Specific Cognitive Trajectories and Dementia Risk: Evidence From the Whitehall II Cohort

**DOI:** 10.1155/jare/1953393

**Published:** 2026-10-28

**Authors:** Sima Toopchiani, Shireen Sindi, Leila Janani, Oliver Robinson

**Affiliations:** ^1^ Ageing Epidemiology (AGE) Research Unit School of Public Health Imperial College London London UK imperial.ac.uk; ^2^ Surgery and Cancer Imperial College London London UK imperial.ac.uk; ^3^ Division of Clinical Geriatrics Center for Alzheimer Research Karolinska Institutet and Karolinska University Hospital Stockholm Sweden ki.se; ^4^ Imperial Clinical Trials Unit School of Public Health Imperial College London London UK imperial.ac.uk; ^5^ Department of Epidemiology and Biostatistics MRC Centre for Environment and Health School of Public Health, Imperial College London London UK

**Keywords:** ageing and dementia, cognition, cognitive performance, decline, sex differences

## Abstract

**Background:**

Evidence on sex differences in cognition and dementia is inconsistent, and it is unclear whether such differences are explained by dementia risk factors.

**Objective:**

To assess (i) sex differences in cognitive decline and dementia risk, (ii) the extent to which modifiable and nonmodifiable risk factors explain these differences and (iii) whether birth cohort and apolipoprotein (APOE)‐Ꜫ4 carrier status modify sex differences in cognitive decline.

**Methods:**

Data from the Whitehall II study were analysed to examine cross‐sectional and longitudinal associations between sex, cognitive decline and dementia risk. Cognitive outcomes included verbal memory and verbal fluency, assessed from Phase 5 onward, with over 16 years of follow‐up. Test scores were standardised using means and standard deviations from the baseline cognitive tests to generate z‐scores. All‐cause dementia was ascertained through linkage to three electronic health record databases.

**Results:**

Among 5920 participants aged 44.8–68.2 years, women demonstrated better baseline performance than men in memory (adjusted mean difference = −0.2, *p* < 0.0001), phonemic fluency (−0.1, *p* = 0.001) and semantic fluency (−0.2, *p* < 0.0001). Men exhibited a steeper decline in semantic fluency (adjusted *β* = −2.6, *p* < 0.0001) and a nominal decline in phonemic fluency (−1.7, *p* = 0.007). Sex differences remained after adjustment for covariates. Dementia incidence did not differ significantly by sex. Birth cohort and APOE‐Ꜫ4 carrier status did not meaningfully modify associations between sex and cognitive outcomes.

**Conclusion:**

Cognitive advantages observed in women in midlife did not translate into lower dementia risk. Further research incorporating biological, psychosocial and life‐course factors, as well as the timing and heterogeneity of underlying disease processes, is needed to better understand sex differences in cognitive ageing and dementia.

## 1. Introduction

Dementia is often characterised as a heterogenous clinical syndrome arising from multiple, often coexisting pathological processes. Although Alzheimer’s disease (AD) makes up 60–70% of all dementia cases in individuals aged ≥ 65 years [1, 2], cerebrovascular disease and other neurodegenerative pathologies, including Lewy body and related pathology, may also contribute to cognitive impairment and dementia in later life [3, 4]. These processes may interact and contribute to differences in the timing and pattern of cognitive decline. Importantly, pathological changes associated with Alzheimer’s disease and related dementias (ADRDs) may accumulate typically 10–20 years before the onset of clinically apparent cognitive impairment [5]. Consequently, longitudinal studies beginning in midlife or older age may capture individuals at different stages of underlying biological disease, even when participants are clinically free of dementia at study entry. Therefore, in the absence of a disease‐modifying cure, efforts to reduce the burden of dementia rely on prevention and early detection, which require a clear understanding of heterogeneity in cognitive ageing and dementia risk. One key dimension of this heterogeneity is sex as approximately two‐thirds of individuals living with dementia are women [6].

Sex differences in cognition exist throughout the lifespan. A growing body of evidence suggests that women perform better on average on specific cognitive domains including memory, processing speed, global cognition and executive function, whereas men tend to perform better on visuospatial tasks [7–9]. The female advantage in memory extends to both the preclinical and mild cognitive impairment (MCI) phase [10], but not the disease stage [11]. Greater resilience to age‐related cognitive decline among women [12–15] may obscure sex differences by contributing to the underestimation of cognitive decline and MCI in women. Studies in the US have shown that sex‐specific norms for verbal memory tests have improved the identification and diagnosis of memory impairment in both men and women [16]. However, these norms are yet to be widely validated or adopted, and it remains unclear whether their application improves or compromises diagnostic accuracy in other cohorts [17].

Longitudinal studies on sex differences in cognitive performance have produced mixed findings. Some report steeper decline in women [18], others in men [19, 20] and some observe no sex differences [10]. Discrepancies may reflect differences in study populations, follow‐up duration, cognitive measures and analytical approaches. These inconsistencies underscore the complexity of sex differences in cognitive ageing and highlight the need for further investigation. Another consideration that is particularly relevant is the long preclinical phase of ADRD when examining longitudinal cognitive trajectories, as observed changes may reflect heterogeneous underlying pathology at various stages of disease rather than a uniform transition from healthy cognitive ageing to dementia.

Sex differences in dementia risk also remain contested. Although the higher prevalence of AD in women is well established [21], evidence for sex differences in incidence is less consistent. While some epidemiological studies have reported a higher incidence of AD among women [22], a pooled meta‐analysis found no significant sex differences in AD risk [23]. Similarly, Huque et al. confirmed that although the global prevalence of AD is higher in women than in men, evidence for sex differences in incidence remains inconclusive as they reported sex differences only among individuals aged 90 years or older [21], which may reflect a survivor effect. Global inconsistencies in reported incidence may depend on time period, sample size and geographical region [24].

The factors underlying sex differences in cognitive performance, decline and dementia risk remain unclear. Advancing age alone does not fully explain the imbalance between the sexes [21, 22, 25]. Sex differences have been reported across several modifiable and nonmodifiable dementia risk factors, including obesity, apolipoprotein (APOE), depression and lifestyle‐related behaviours, suggesting that differential exposures to these factors across the life course may contribute to observed differences in cognitive outcomes and dementia risk [26]. Gendered life experiences such as differential access to education can shape cognitive development, ageing and dementia risk [27]. These risk factors may also vary across birth cohorts, reflecting differences in societal and environmental exposures across the life course. Birth cohorts reflect societal context shaped by significant historical events that influence opportunities and experiences [28]. Later‐born cohorts tend to show better performance on a range of cognitive tasks, even after accounting for education [29, 30]. In addition, cohort effects have been observed in cognitive trajectories and rates of decline, although findings vary across populations and study designs [31–34].

Together, these findings underscore the importance of social determinants and life‐course factors in understanding sex differences in cognitive outcomes. However, the contribution of dementia risk factors to these differences remains unclear. Addressing this gap requires large, well‐characterised longitudinal cohorts with repeated cognitive assessments and detailed risk factor data. Furthermore, previous studies have examined global cognitive decline as the primary outcome, which is an important endpoint for understanding overall brain health deterioration. Examining individual cognitive domains may provide additional insight into these heterogenous processes, as different domains may show distinct patterns of change between men and women. Understanding whether these domain‐specific trajectories differ between men and women may therefore provide some understanding into potential differences in the timing or pattern of cognitive ageing and subsequent dementia risk. The Whitehall II study provides a valuable opportunity to examine these questions and clarify the extent to which sex differences in domain‐specific cognitive trajectories and dementia risk are explained by differential exposure to established AD‐dementia risk factors.

This study aims to investigate the association between sex, cognitive function and dementia risk utilising up to 16 and 18 years of follow‐up, respectively, in the Whitehall II study of London‐based civil servants. Specifically, we aimed to (i) quantify sex differences in memory and fluency at baseline and over time, (ii) examine sex differences in dementia risk, (iii) determine the extent to which dementia risk factors explain the observed sex differences and (iv) assess whether nonmodifiable risk factors, including age, birth cohort and APOE‐Ꜫ4 carriage, modify the association between sex and cognition.

## 2. Methods

### 2.1. Study Setting and Population

Whitehall II is an ongoing prospective cohort study, established in 1985, of civil servants (men and women) aged 35–55 years working in London‐based offices [35] from 20 departments. Civil servants were invited to participate in the study by letter, and a response rate of 73% was achieved [36]. The cohort consisted of 10,308 participants, including 3413 women and 6895 men.

The screening visits were conducted at a clinic and included a clinical examination and a detailed questionnaire on their health behaviours, socioeconomic status (SES), work environment and psychosocial factors [35]. Individuals in certain job grades that were too low to allow meaningful comparisons, as well as those in active service or who had only recently joined the civil service, were excluded in the original Whitehall II study design [35]. Participants have been followed through repeated assessment phases approximately every 4‐5 years. Cognitive assessments were first introduced in Phase 5 (1997–99). Participants subsequently completed cognitive testing during Phases 7 (2002–04), 9 (2007–09), 11 (2012‐13) and 12 (2015‐16). For the present study, data from Phase 5 onwards to Phase 12 were used.

The original Whitehall II study has been approved by relevant ethics committees since 1985, including most recently by the UCL Hospital Committee on the Ethics of Human Research (reference 85/0938). The study was conducted in accordance with the Declaration of Helsinki. All participants who had a clinical examination were asked to give written informed consent, and this was renewed at each contact. The present study involved secondary analysis of existing Whitehall II data and did not involve new data collection or participant contact; therefore, no additional ethics approval was required.

### 2.2. Study Sample

Of 10,308 participants in the Whitehall II study, 306 (3%) had passed away, and 752 (7.3%) had withdrawn consent before the start of Phase 5 (Figure 1). Among the 9250 in Phase 5, those with missing data on baseline age (0.2%) or cognitive measures (memory: 41.8%; fluency: 41.7%) were excluded. The analytical sample therefore included 5920 participants at baseline. The follow‐up cohort consisted of 5047 participants, excluding 873 participants without at least one subsequent cognitive assessment.

**FIGURE 1:**
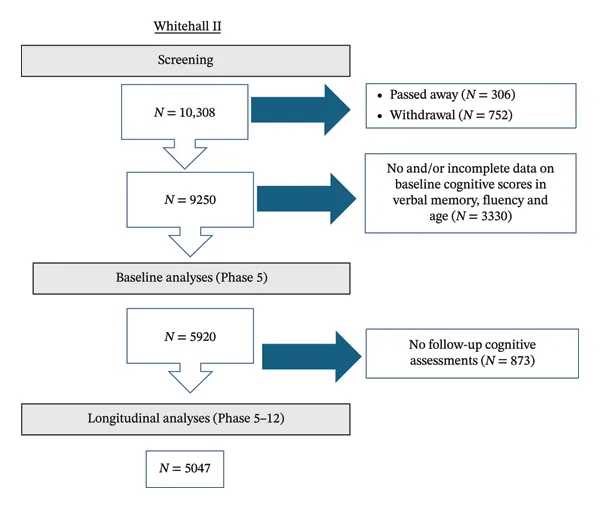
CONSORT diagram showing the number of participants included in the baseline and follow‐up analysis of the Whitehall II Study.

### 2.3. Outcome

#### 2.3.1. Cognition

Following the introduction of the cognitive test battery at Phase 5, a subsample of 556 participants was re‐examined within three months to assess test–retest reliability, which was found to be good (range: 0.60–0.89) [36]. Previous work has suggested that tests of memory and verbal fluency are appropriate measures of cognitive function and abilities in populations younger than those typically included in studies of cognitive ageing [36].

Baseline was defined as the first cognitive assessment available for participants (Phase 5) [37] included in the present analysis. Participants were free from clinically diagnosed dementia at baseline; however, the absence of clinical dementia does not preclude the presence of preclinical neurodegenerative or vascular pathology. Data from Phases 5, 7, 9, 11 and 12 were used to examine cognitive outcomes over time.

*Short-term memory* was assessed using immediate free recall. Participants listened to a list of 20 one‐two syllable words at 2‐second intervals on a tape and then were asked to recall in writing as many as possible within two minutes (score = out of 20). Two tests were used to assess verbal fluency: phonemic and semantic. Verbal fluency is a cognitive domain that can be used to assess an individual’s ability to retrieve information from memory, and tests of verbal fluency evaluate the ability to retrieve specific information within restricted search parameters [38]. *Phonemic fluency* was assessed via ‘S’ words, and subjects were given 1 minute to recall as many words beginning with ‘S’. *Semantic fluency* was assessed via an animal naming task, and participants were given 1 minute to write as many animal names as possible. In both tests of verbal fluency, scores were out of 35, with higher scores reflecting better performance.

#### 2.3.2. Dementia

Three databases were integrated for ascertainment of all‐cause dementia [39]: the national Hospital Episode Statistics database (contains clinical diagnoses from inpatient, outpatient and emergency departments), the Mental Health Services Data Set (contains dementia diagnosis from inpatient, outpatient and community mental health services) and the mortality register. The record linkage occurred until 31^st^ March 2015, using international classification of diseases 10^th^ edition codes F00, F01, FO2, FO3, FO5.1, G30, G31.0, G31.1 and G31.8 identified cases of dementia and the tracing exercise used the NHS identification number issued to each resident in the UK [39]. The date of dementia diagnosis at the first record in any of the three databases was set at the date of dementia diagnosis.

#### 2.3.3. Covariates

Covariates were collected through a self‐administrated postal questionnaire completed prior to the screening examination visit. Questionnaires were checked for completeness during the clinic visit, and missing information was obtained through interviewer clarification. Sociodemographic variables included age (years), sex (male or female) and ethnicity. Ethnicity was categorised as White or Other, where Other included Black‐Caribbean, Indian, Chinese, Black‐African, Pakistani, Black‐Other, Bangladeshi and Other. Education was categorised as lower secondary school (no qualifications/O‐Levels), high secondary school (A‐levels) and university degree. Marital status was categorised as married/cohabiting or single, occupation was categorised as clerical/support, administrative, or professional/executive and annual household income was categorised as follows: < £20k, < 40k, < 60k and ≥ £60k.

Lifestyle behaviours included smoking status which was categorised as current, ex‐smoker or nonsmoker, alcohol consumption (14 > or ≤ 14 units per week) as per the United Kingdom (UK) recommended guidelines [40], frequency of fruit and vegetable consumption (5 > or ≤ 5 items per day) and sleep duration which was categorised as participants who slept ≤ 6 h, 7–9 h ≥ or > 9 h per night. Physical activity was categorised as meeting the ‘recommended physical activity’ defined using the World Health Organization (WHO) criteria of moderate to vigorous activity for at least 2.5 h per week [41].

Health‐related variables such as hypertension, diabetes, stroke and cardiovascular disease (CVD) and body mass index (BMI). Hypertension was defined as measured systolic blood pressure ≥ 140 mmHg or measured diastolic blood pressure ≥ 9 mmHg or current use of antihypertensive drugs. Diabetes was defined as measured fasting glucose ≥ 7 mmol/L, diagnosed by doctor or the use of diabetes drugs. Stroke and CVD were defined as doctor diagnosed (verified in patient medical records). BMI was calculated using body weight (kg) and height (cm), measured during the clinical examination.

Symptoms of anxiety and depression were assessed by the Whitehall II study team using the 30‐item General Health Questionnaire (GHQ) [42]. Depressive symptoms were measured as a total score and ranged from 0 to 12. A total score of ≥ 4 was used to define the presence of depressive symptoms [42]. A total score of ≥ 8 was used to define anxiety cases [43]. Cancer diagnosis was self‐reported as part of the GHQ. Self‐reported cases of cancer were cross‐checked with medical records to ensure accuracy.

#### 2.3.4. APOE Genotyping

DNA was extracted from whole blood samples, which were drawn at the 1997–1999 clinical examination (Phase 5). Two TaqMan assays (Rs2429358 and Rs7412, Assay‐On‐Demand, Applied Biosystems) were used and run on a 7900HT analyser (Applied Biosystems), and APOE genotypes were indicated by the Sequence Detection Software Version 2.0 (Applied Biosystems) [44, 45]. Participants were then categorised based on their genotype as ‘carrier’ if they carried one or two *ε*4 alleles and or ‘noncarrier’ if they carried none.

### 2.4. Statistical Analysis

Participant characteristics were summarised separately for men and women using chi‐square tests for categorical variables and *t*‐tests for continuous variables, with Cohen’s d used to estimate effect sizes for differences in cognitive measures. We standardised all continuous measures of cognition (verbal memory and verbal fluency) using the mean and SD from the baseline cognitive tests. We conducted four sets of analyses, described below.

#### 2.4.1. Association Between Sex and Baseline Cognitive Function

Cross‐sectional analysis was based on 5920 participants. Using linear regression models, we first examined whether there was an association between sex and cognitive function at baseline adjusted for age. We then adjusted the analyses using three additional models: Model 1 included ethnicity (white/other), Model 2 additionally adjusted for socioeconomic factors (education, annual household income, marital status and occupation) and Model 3 additionally included vascular (BMI, hypertension, diabetes, stroke and CVD) and lifestyle factors (alcohol consumption, smoking status, fruit and vegetable intake, sleep duration and physical activity) and other comorbidities (cancer, depression and anxiety). The regression coefficient (β) represents the average difference in mean cognitive scores (reference category: women).

#### 2.4.2. Association Between Sex and Cognitive Function Over Time

This analysis included 5047 participants with data on baseline cognitive function and at least one follow‐up assessment. Linear mixed effects models were used to examine the association between sex and cognitive trajectories from 1995–97 to 2015‐16. Time (years since baseline) was modelled as a continuous variable, and an interaction term between sex and time (sex x time) was included to assess whether the rate of cognitive change differed by sex. Random intercepts and slopes were fitted in the models to account for repeated measures and varying follow‐up durations. Models were fully adjusted models for the same covariates as described above. The regression coefficient (β) for the sex × time interaction represents the difference in mean cognitive scores per year (reference category: women).

#### 2.4.3. Association Between Sex and Dementia

The association between sex and incident dementia was assessed from Phase 5 to Phase 12. This analysis included 4154 participants. Those with dementia at Phase 5 (baseline) were excluded, and 289 incident dementia cases were identified during follow‐up. Cox proportional hazards (PH) models were used to estimate hazard ratios (HRs), with age at dementia diagnosis or age at the end of follow‐up as the underlying timescale. The PH assumption was assessed using Schoenfeld residuals and was deemed satisfactory. Models were fully adjusted for the same covariates as described above.

#### 2.4.4. Subgroup Analyses

Prespecified subgroup analyses were conducted according to two nonmodifiable risk factors: birth cohort (*N* = 4826) and APOE‐Ꜫ4 carriage (*N* = 4455). Participants were stratified by birth cohort (1935–1944 vs. 1945–1954) and by APOE‐ε4 carrier status (noncarrier, heterozygote and homozygote). Linear mixed‐effects models were fitted to estimate the main effects of sex and the sex × time interaction on cognitive trajectories. Effect modification by birth cohort and APOE‐*ε*4 carrier status was assessed by fitting models including three‐way interaction terms (sex x birth cohort x time and sex x APOE x time). Wald tests were used to assess the significance of the interaction terms. Models examining APOE‐*ε*4 were adjusted for baseline age, whereas models examining birth cohort were fully adjusted for the same covariates described above.

Missing data were handled using multiple imputation. Ten imputed datasets were generated, and imputation was performed for all variables with missing data included in the analysis. Supporting Table S1 displays the number (N) and percentage (%) of missing variables. The analyses were conducted separately in each imputed dataset, and the results were combined using Rubin’s rules to obtain pooled coefficients and standard errors. Patterns of missingness were explored using heatmaps and cross‐tabulations against key sociodemographic and clinical variables, suggesting that the data were missing at random (MAR).

All statistical analyses were conducted using Stata (Version 18; StataCorp). A two‐sided *p* value of < 0.05 was considered the general threshold for statistical significance. The Bonferroni‐corrected threshold was calculated as *p* < 0.005 (0.05/11) applied to account for multiple comparisons across the three cognitive tests and three interaction tests.

## 3. Results

### 3.1. Study Population

We included 5920 of 10,308 participants of the original cohort. Of 5920 participants assessed at baseline, the mean (SD) age was 55.6 (6.0) years (Table 1). Among all baseline participants, 71.2% were male, 28.8% were female and nearly all participants (91.4%) were white. Compared with women, men were more likely to be better educated and hold an undergraduate/postgraduate degree (*p* < 0.0001). Men also reported higher annual household incomes (≥£60,000: 14% vs. 9.6%), were more likely to be married/cohabiting (81.7%) and employed in higher occupational positions including professional/executive (46.3%) roles while women were more likely to be employed in clerical/supportive roles (32%). Furthermore, men were more likely to drink > 14 units per week (40.8%), consume < 5 pieces of fruit and vegetables per day (63.1%) and meet the WHO guidelines for physical activity (26.1%). Women had higher BMI (*p* = 0.002), were more likely to have CVD (17.1%), sleep ≤ 6 h a night (42.6%) and have cancer (5%), depression, (13.8%) and anxiety (12.8%). Smoking status differed by sex, with higher proportions of smokers (11.3% vs. 8.8%) and nonsmokers (54.9% vs. 47.2%) among women, and a higher proportion of ex‐smokers (31.5% vs. 43%) among men. Men had significantly lower scores than females in phonemic fluency (*p* = 0.05), but men scored significantly higher in semantic fluency (*p* < 0.0001), and no sex differences were observed in memory.

**TABLE 1:** Baseline characteristics by sex in the analytical sample.

Cohort	Baseline cohort				Longitudinal cohort			
	All	Female	Male	*p*	All	Female	Male	*p*
*N*	5920	1703 (28.8%)	4217 (71.2%)		5047	1398 (27.7%)	3649 (72.3%)	

*Demographics*								
Age (y), mean (SD)	55.6 (6.0)	55.9 (6.0)	55.5 (6.0)	0.007	55.4 (6.0)	55.5 (6.0)	55.3 (6.0)	0.3
Ethnicity, *n* (%)				< 0.0001				< 0.0001
White	5413 (91.4%)	1464 (86%)	3949 (93.6%)		4655 (92.2%)	1214 (86.8%)	3441 (94.3%)	
Other	506 (8.5%)	239 (14%)	267 (6.3%)		391 (7.7%)	184 (13.2%)	207 (5.7%)	

*Socioeconomic factors*								
Education, *n* (%)				< 0.0001				< 0.0001
No school qualifications/O‐Levels	2110 (35.6%)	851 (50%)	1259 (29.9%)		1721 (34.1%)	658 (47.1%)	1063 (29.1%)	
A‐Levels	1594 (26.9%)	344 (20.2%)	1250 (29.6%)		1405 (27.8%)	309 (22.1)	1096 (30%)	
Undergraduate/postgraduate degree	2108 (35.6%)	456 (26.8%)	1652 (39.2%)		1845 (36.6%)	397 (28.4%)	1448 (39.7%)	
Household annual income, *n* (%)				< 0.0001				< 0.0001
< £20, 000	1089 (18.4%)	506 (29.7%)	583 (13.8%)		860 (17%)	398 (28.5%)	462 (12.7%)	
< £40, 000	1955 (33%)	516 (30.3%)	1439 (34.1%)		1688 (33.4%)	436 (31.2%)	1252 (34.3%)	
< £60, 000	1157 (19.5%)	216 (12.7%)	941 (22.3%)		1040 (20.6%)	193 (13.8%)	847 (23.2%)	
≥ £60,000	755 (12.8%)	164 (9.6%)	591 (14%)		667 (13.2%)	143 (10.2%)	524 (14.4%)	
Occupation, *n* (%)				< 0.0001				< 0.0001
Clerical/support	756 (12.8%)	545 (32%)	211 (5%)		551 (10.9%)	408 (29.2%)	143 (3.9%)	
Administrative	2535 (42.8%)	351 (20.6%)	2184 (51.8%)		2244 (44.5%)	310 (22.2%)	1934 (53%)	
Professional/executive	2577 (43.5%)	789 (46.3%)	1788 (42.4%)		2209 (43.8%)	666 (39.1%)	1543 (42.3%)	
Marital status, *n* (%)				< 0.0001				< 0.0001
Married/cohabiting	4427 (74.8%)	982 (57.7%)	3445 (81.7%)		3809 (75.5%)	805 (57.6%)	3004 (82.3%)	

*Cardiometabolic factors*								
BMI (units), mean (SD)	26.1 (4.0)	26.4 (4.9)	26.0 (3.5)	0.002	26.1 (3.9)	26.4 (4.9)	26 (3.5)	0.002
Hypertension, *n* (%)	1090 (18.4%)	312 (18.3%)	778 (18.4%)	0.9	858 (17%)	238 (17%)	620 (17%)	1
Diabetes, *n* (%)	198 (3.3%)	57 (3.3%)	141 (3.3%)	0.9	148 (2.9%)	43 (3.1%)	105 (2.9%)	0.7
Stroke, *n* (%)	< 5 (< *n*%)	< 5 (< *n*%)	< 5 (< *n*%)	0.2	5 (0.1%)	0 (0%)	5 (0.1%)	0.2
CVD, *n* (%)	928 (15.7%)	292 (17.1%)	636 (15.1%)	0.04	732 (14.5%)	223 (16%)	509 (13.9%)	0.07

*Lifestyle factors*								
Alcohol (units per week), *n* (%)				< 0.0001				< 0.0001
≤ 14	3757 (63.5%)	1371 (80.5%)	2386 (56.6%)		3188 (63.2%)	1144 (81.8%)	2044 (56%)	
> 14	1982 (33.5%)	261 (15.3%)	1721 (40.8%)		1716 (34%)	207 (14.8%)	1509 (41.4%)	
Smoking status, *n* (%)				< 0.0001				< 0.0001
Nonsmoker	2926 (49.4%)	935 (54.9%)	1991 (47.2%)		2518 (49.9%)	779 (55.7%)	1739 (47.7)	
Ex‐smoker	2349 (39.7%)	536 (31.5%)	1813 (43%)		2039 (40.4%)	452 (32.3%)	1587 (43.5%)	
Smoker	563 (9.5%)	192 (11.3%)	371 (8.8%)		426 (8.4%)	141 (10.1%)	285 (7.8%)	
Diet (consumption of fruit and vegetables), *n*(%)				< 0.0001				< 0.0001
< 5 (per day)	3509 (59.3%)	849 (49.9%)	2660 (63.1%)		2957 (58.6%)	679 (48.6%)	2278 (62.4%)	
≥ 5 (per day)	2230 (37.7%)	781 (45.9%)	1449 (34.4%)		1949 (38.6%)	669 (47.9%)	1280 (35.1%)	
Sleep (hours per night), *n* (%)				0.002				0.002
≤ 6 h	2380 (40.2%)	725 (42.6%)	1655 (39.2%)		1995 (39.5%)	585 (41.8%)	1410 (38.6%)	
≤ 9 h	3305 (55.8%)	890 (52.3%)	2415 (57.3%)		2861 (56.7%)	748 (53.5%)	2113 (57.9%)	
> 9 h	74 (1.3%)	29 (1.7%)	45 (1.1%)		62 (1.2%)	24 (1.7%)	38 (1%)	
Physical activity (WHO guidelines), *n* (%)				< 0.0001				< 0.0001
No	4415 (74.6%)	1391 (81.7%)	3024 (71.7%)		3754 (74.4%)	1147 (82.1%)	2607 (71.4%)	
Yes	1360 (23%)	260 (15.3%)	1100 (26.1%)		1183 (23.4%)	220 (15.7%)	963 (26.4%)	

*Comorbidities and psychological factors*								
Cancer, *n* (%)	186 (3.1%)	85 (5%)	101 (2.4%)	< 0.0001	157 (3.1%)	69 (4.9%)	88 (2.4%)	< 0.0001
Depression, *n* (%)	713 (12%)	235 (13.8%)	478 (11.3%)	0.005	585 (11.6%)	186 (13.3%)	399 (10.9%)	0.01
Anxiety, *n* (%)	541 (9.1%)	218 (12.8%)	323 (7.7%)	< 0.0001	454 (9%)	182 (13%)	272 (7.5%)	< 0.0001

*Cognitive performance*								
Memory, mean (SD)	6.9 (2.4)	6.9 (2.7)	6.9 (2.3)	0.3	7.0 (2.6)	6.9 (2.4)	6.9 (2.3)	0.1
Phonemic fluency, mean (SD)	10.0 (5.8)	10.3 (6.4)	9.9 (5.5)	0.05	10.0 (5.5)	10.1 (5.9)	9.9 (5.3)	0.2
Semantic fluency, mean (SD)	16.4 (4.2)	15.8 (4.6)	16.6 (4.0)	< 0.0001	16.6 (4.1)	16.1 (4.5)	16.8 (4.0)	< 0.0001

*Note:* Independent *t*‐tests were used for continuous variables and chi‐square tests for categorical variables. Continuous variables as mean (SD) and categorical variables are presented as *N* (%). Cell counts below 5 have been suppressed and reported as < *n*.

In the longitudinal cohort (Table 1), data from 5047 participants were available and similar patterns in sex differences in baseline characteristics were observed, but the sex difference in age was no longer observed. Similarly, sex differences in phonemic fluency were no longer observed but remained in semantic fluency (*p* < 0.0001).

Cohen’s d indicated negligible sex differences for memory and phonemic fluency, but a small effect size favouring men for semantic fluency (Table 2). Cohen’s U3 values showed that approximately half of women scored above the male mean for memory and phonemic fluency, whereas fewer women did so for semantic fluency.

**TABLE 2:** Effect sizes (Cohen’s d and U3) for memory and fluency at baseline, using women as the reference group.

Cognitive domain	Cohen’s d	Cohen’s U3
Memory	0.0291	0.51
Phonemic fluency	0.0591	0.52
Semantic fluency	−0.2	0.42

### 3.2. Sex Differences in Baseline Cognitive Performance

Table 3 presents the results of cross‐sectional analyses examining sex differences in baseline cognitive performance in verbal memory and fluency. Models were progressively adjusted for covariates: Model 1 includes demographic variables, Model 2 additionally adjusts for socioeconomic factors and Model 3 further adjusts for cardiometabolic and lifestyle factors, as well as other prevalent diseases.

**TABLE 3:** Cross‐sectional associations of sex with cognitive performance in memory and fluency at baseline (*N* = 5920), based on progressively adjusted multiple regression models (Models 1–3).

Model no.	Difference in mean scores men compared to women (β) (95% CI)	*p*
*Memory*		
Ref: women	—	—
Model 1	−0.09 (−0.1, −0.03)	0.001^†^
Model 2	−0.2 (−0.3, −0.2)	< 0.0001^†^
Model 3	−0.2 (−0.3, −0.2)	< 0.0001^†^

*Phonemic fluency*		
Model 1	−0.07 (−0.1, −0.009)	0.02^∗^
Model 2	−0.1 (−0.2, −0.05)	0.001^†^
Model 3	−0.1 (−0.2, −0.05)	0.001^†^

*Semantic fluency*		
Model 1	0.1 (0.05, 0.2)	< 0.0001^†^
Model 2	−0.2 (−0.2, −0.1)	< 0.0001^†^
Model 3	−0.2 (−0.2, −0.09)	< 0.0001^†^

*Note:* Model 1: baseline age and ethnicity, Model 1 + 2: education, household annual income, occupation and marital status and Model 1, 2 + 3: BMI, hypertension, diabetes, stroke, CVD, alcohol consumption, smoking status, diet, sleep, physical activity, cancer, depression and anxiety. Nominal significance threshold: *p* < 0.05 (^∗^). Bonferroni‐corrected significance threshold: *p* < 0.005 (^†^). Values meeting the Bonferroni‐corrected threshold are marked^†^; those meeting only the nominal threshold are marked^∗^.

Adjusting for baseline age and ethnicity, sex differences favoured women in memory (mean difference between men and women = −0.09, 95% CI: −0.1, −0.03) and favoured men in semantic fluency (mean difference = 0.1; 95% CI: 0.05, 0.2). In phonemic fluency, sex differences were in favour of women (mean difference = −0.07; 95% CI: −0.1, −0.009), and this effect reached nominal significance but did not survive correction for multiple testing. Additional adjustment for socioeconomic factors and marital status (Model 2) found men scored worse in all cognitive tests including memory (mean difference = −0.2; 95% CI: −0.3, −0.2), phonemic (mean difference = −0.1; 95% CI: −0.2, −0.05) and semantic fluency (mean difference = −0.2; 95% CI: −0.2, −0.1). The magnification of the association conveys an underestimation of sex differences in these cognitive domains due to the (negative) confounding effects of socioeconomic factors, i.e., educational attainment, occupation, annual household income and marital status. However, the additional adjustment of cardiometabolic (including BMI, hypertension, diabetes, stroke and CVD), lifestyle factors and other prevalent diseases (Model 3) had no additional effect on the association between sex and cognitive performance.

Results from the imputed (Table 3) and nonimputed complete‐case analysis (Supporting Table S2) assessing the association between sex and cognitive performance at baseline were similar.

### 3.3. Sex Differences in Cognitive Decline

Figure 2 presents the projected mean change (per year) in memory, phonemic fluency and semantic fluency. Sex differences were observed in both fluency measures: men showed faster decline in phonemic fluency (annual change in mean difference = −1.7, 95% CI: −2.9, −0.5, *p* = 0.007), which did not survive Bonferroni correction, and in semantic fluency (annual change in mean difference = −2.6, 95% CI: −4.1, −1.2, *p* < 0.0001) over 16 years of follow‐up, in fully adjusted models (Table 4). No sex differences were observed in change in memory over time. Supporting Table S3 shows progressive adjustment for covariate sets (social, vascular, lifestyle and prevalent disease factors), with the results remaining essentially unchanged compared to the basic model.

**FIGURE 2:**
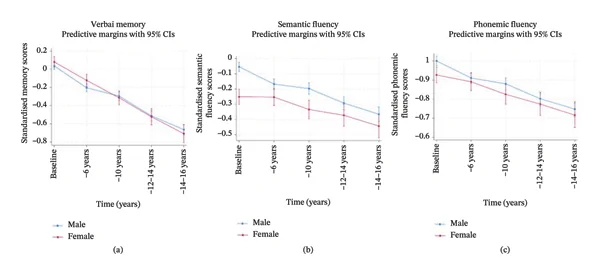
(a–c) Projected mean change (per year) in verbal memory, phonemic and semantic fluency over 16 years with 95% confidence error bars over time by sex. Linear mixed models were used to assess change in participants’ cognitive performance over time (per year), by modelling the interaction between sex and time on change in cognitive trajectories. Model was adjusted for baseline age, ethnicity, education, occupation, household annual income, marital status, BMI, CVD, hypertension, diabetes, stroke, alcohol consumption, diet, physical activity, cancer, depression and anxiety, *N* = 5047.

**TABLE 4:** Mixed model analyses assessing the interaction between sex and time on annual and 5‐year change in memory and fluency, adjusted for baseline covariates (*N* = 5047).

Fully adjusted model	Change in mean scores per year (β) (95% CI)	*p*	Change in mean scores 5 years (β) (95% CI)	*p*
*Memory*				
Ref: women	—	—	—	—
1. Main effect: male^1^	−0.2 (−0.3, −0.2)	< 0.0001^†^	−0.2 (−0.3, −0.2)	< 0.0001^†^
2. Interaction between male and time^2^	0.3 (−1.4, 2.1)	0.7	1.7 (−7.2, 10.6)	0.7

*Phonemic fluency*				
Ref: women	—	—	—	—
1. Main effect: male^1^	−0.1 (−0.2, −0.09)	< 0.0001^†^	−0.1 (−0.2, −0.09)	< 0.0001^†^
2. Interaction between male and time^2^	−1.7 (−2.9, −0.5)	0.007^∗^	−8.4 (−14.6, −2.3)	0.007^∗^

*Semantic fluency*				
Ref: women	—	—	—	—
1. Main effect: male^1^	−0.09 (−0.2, −0.03)	0.003^†^	−0.09 (−0.2, −0.03)	0.003^†^
2. Interaction between male and time^2^	−2.6 (−4.1, −1.2)	< 0.0001^†^	−13.2 (−20.3, −6.1)	< 0.0001^†^

*Note:* Nominal significance threshold: *p* < 0.05 (^∗^). Bonferroni‐corrected significance threshold: *p* < 0.005 (^†^). Values meeting the Bonferroni‐corrected threshold are marked^†^; those meeting only the nominal threshold are marked^∗^. Adjusted for baseline age, ethnicity, education, occupation, household annual income, marital status, BMI, CVD, hypertension, diabetes, stroke, alcohol consumption, diet, physical activity, cancer, depression and anxiety.

^1^Difference in mean scores, men compared to women.

^2^Change in mean scores per year in men compared to women.

Results from the imputed longitudinal analysis (Table 4) were similar to the complete case analysis (Supporting Table S4).

### 3.4. Sex Differences in Incident All‐Cause Dementia

Over an average follow‐up of 9.4 years, no significant difference in all‐cause dementia risk was observed between men and women in the Whitehall II cohort (Table 5). Although men initially showed a slightly higher risk after adjusting for baseline age and ethnicity (Model 1), this association attenuated with additional adjustment for socioeconomic factors (Model 2). After further adjustment for cardiometabolic and lifestyle factors as well as cancer, depression and anxiety (Model 3), the association reversed but remained nonsignificant.

**TABLE 5:** Association of sex with dementia (*N* = 4154).

Model no.	*N* (cases/total)	Hazard ratio (95% CI)	*p*
Model 1	(289/4154)	1.1 (0.9, 1.4)	0.4
Model 2	(289/4154)	1.0 (0.7, 1.3)	0.9
Model 3	(289/4154)	0.9 (0.7, 1.3)	0.6

*Note:* Model 1: baseline age and ethnicity, Model 1 + 2: education, household annual income, occupation and marital status and Model 1, 2 + 3: BMI, hypertension, diabetes, stroke, CVD, alcohol consumption, smoking status, diet, sleep, physical activity, cancer, depression and anxiety.

### 3.5. Exploratory Analyses

#### 3.5.1. Effect Modification and Subgroup Estimates by Birth Cohort Assessing the Association Between Sex and Cognitive Trajectories Over 10 Years

The three‐way interaction between sex, time and birth cohort on cognitive performance in semantic fluency in the 1935–44 birth cohort was nominally significant (*p* = 0.01) in the fully adjusted model but did not survive Bonferroni correction. This suggests that the magnitude of sex differences in rates of decline in semantic fluency may differ across birth cohorts, with evidence in the 1935–44 cohort of faster decline in men (annual change in mean difference: −2.8, 95% CI: −4.9, −0.7, *p* = 0.01) compared to women (Figure 3). In contrast, in the 1945–54 cohort, there was no evidence of a sex difference in rates of decline in semantic fluency (annual change in mean difference: −1.8, 95% CI: −3.9, 0.3, *p* = 0.09).

**FIGURE 3:**
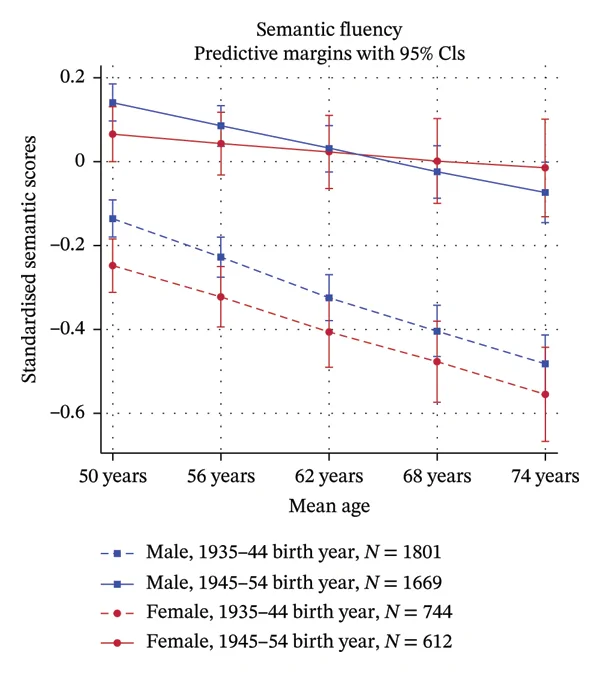
Projected mean change (per year) in semantic fluency over time by sex and birth cohort with 95% confidence error bars. Linear mixed models were used to assess change in participant’s cognitive performance over time (per year), by modelling the interaction between sex, time and birth cohort on change in semantic fluency. X‐axis represents mean age at each time point. Model was adjusted for ethnicity, education, occupation, household annual income, marital status, BMI, CVD, hypertension, diabetes, stroke, alcohol consumption, diet, physical activity, cancer, depression and anxiety, *N* = 4826.

The three‐way interaction between sex, time and birth cohort was not statistically significant for memory (1935‐44: *p* = 0.5; 1945‐54: *p* = 0.9) or phonemic fluency (1935‐44: *p* = 0.1; 1945‐54: *p* = 0.1), indicating no evidence of effect modification by birth cohort. Sex × time interactions were therefore examined within each birth cohort (Supporting Table S5). In phonemic fluency, estimates suggested slightly larger sex differences in the 1935–44 cohort (annual change in mean difference: −1.8, 95% CI: −3.5, −0.1) compared with the 1945–55 cohort (vs. −1.5, 95% CI: −2.6, −0.4), although these differences did not survive correction for multiple testing. A similar pattern was observed for semantic fluency, with larger estimates in the 1935–44 cohort (−3.1, 95% CI: −5.0, −1.2) than the 1945–54 cohort (−2.3, 95% CI: −3.6, −0.1), with both comparisons reaching the Bonferroni‐corrected threshold (*p* = 0.002 and *p* = 0.001). No evidence of sex differences was observed for memory.

#### 3.5.2. Effect Modification and Subgroup‐Specific Estimates by APOE‐Ꜫ4 Carrier Status Assessing the Association Between Sex and Cognitive Trajectories Over 16 Years

The three‐way interaction between sex, time and APOE‐Ꜫ4 heterozygote status on cognitive performance in phonemic fluency showed a nominal association (*p* = 0.05) that did not survive Bonferroni correction. This suggests a possible variation in the association between sex and cognitive change over time in phonemic fluency by APOE‐Ꜫ4 carrier status (Supporting Figure S1 and Table S6, Model 3). Among noncarriers, men demonstrated lower phonemic fluency scores over time compared with women (mean difference in annual change: −2.0, 95% CI: −3.5, −0.5), whereas little sex difference was observed among APOE‐Ꜫ4 heterozygotes (mean difference in annual change: 0.3, 95% CI: −2.5, 3.0). The corresponding three‐way interaction estimate was 3.3 (95% CI: 0.05, 6.6).

The three‐way interaction between sex, time and APOE‐Ꜫ4 carriage was not statistically significant in memory and semantic fluency (Table S6).

## 4. Discussion

In analysis of almost 6000 London‐based civil servants, we found that women demonstrated an advantage in memory and verbal fluency performance. Interestingly, adjustment for baseline covariates strengthened the female advantage in memory and attenuated the initial male advantage observed in semantic fluency, suggesting negative confounding by sociodemographic factors. Sex differences remained favourable to women longitudinally. Despite these sex differences in cognitive performance and decline, no overall association between sex and dementia incidence was observed.

Observed sex differences in cognition at baseline are consistent with previous studies reporting domain‐specific differences in cognitive performance and decline [46, 47]. Studies of verbal memory have consistently found that women perform better than men [48–52]. The underlying mechanisms for these sex differences remain unclear. One explanation is the neuroprotective effect of oestrogen [53], which may support hippocampal function and contribute to women’s superior memory performance during midlife. Furthermore, cognitive reserve theory has been proposed as a potential explanation for sex differences in cognition and cognitive decline [54–56]; however, given that women in this sample had lower levels of education and occupational attainment, this mechanism may not fully account for the observed differences. Another explanation could be varying levels of underlying pathology which in most longitudinal epidemiological studies was not considered.

After adjusting for baseline demographic, socioeconomic, lifestyle, cardiometabolic and health factors (including depression, anxiety and cancer), women scored higher in both measures of fluency. This finding aligns with a study by Acevedo and colleagues [57] reporting a female advantage in semantic fluency in a sample of 702 cognitively intact individuals aged 50–90 years and with a recent meta‐analysis of 355,173 participants that found a small but robust female advantage in phonemic fluency [51]. However, other studies did not observe sex differences in both tests of fluency [58–60], although these included participants at much younger ages than the Whitehall II study.

Prior longitudinal studies examining sex differences in cognitive ageing have reported mixed findings. Consistent with the present results, a 10‐year study of 625 cognitively healthy adults found a female advantage in episodic and verbal memory at baseline but no sex differences in memory decline over time [53]. Similarly, a systematic review concluded that rates of cognitive decline between ages 60 and 80 were more strongly influenced by contextual and cultural factors than by sex alone [61]. In contrast, a 12‐year longitudinal study of 7485 adults reported a greater memory decline in women despite higher baseline performance [62]. However, this effect was only observed in participants older than > 76 years, while sex differences remained stable between ages 20–76 years.

In contrast to the findings for memory, sex differences were observed in verbal fluency in the present study. Men showed faster decline in semantic fluency, which survived Bonferroni correction, while faster decline in phonemic fluency reached only nominal significance. These findings are broadly consistent with studies reporting steeper decline in men across some cognitive domains. For example, the Baltimore Longitudinal Study of Ageing found faster decline in men in perceptuomotor speed and visuospatial ability, although sex differences in verbal fluency were not reported [19]. Similarly, a 27‐year longitudinal study reported higher rates of cognitive impairment in men, whereas women exhibited faster decline in executive function after cognitively impaired participants were excluded [63]. Together, these findings suggest that women may show greater resilience to certain aspects of age‐related cognitive decline in cognitively health populations.

Some studies, however, reported steeper decline in women [64]. These discrepancies may reflect differences in cohort composition, follow‐up duration, cognitive health, age distribution and demographic diversity. Methodological differences may also contribute, including semantic categories used in verbal fluency tasks. For example, one study reported steeper decline in category fluency in men [19], whereas another found no sex differences when using only the ‘animals’ category [65]. The timing of cognitive decline may also be important, as individuals may enter longitudinal studies at different stages of cognitive ageing or underlying neurodegenerative disease. Future studies could examine age at onset of clinically significant cognitive change or changes in cognitive slope to determine whether sex differences vary according to the timing of decline. Overall, these findings suggest that sex differences in cognitive ageing are domain‐specific and reflect a complex interplay of demographic, methodological, contextual and biological factors.

In both the cross‐sectional and longitudinal analyses, differences in cognitive performance and decline between men and women remained after adjustment for covariates. Consistent with previous studies, adjustment for socioeconomic factors influenced the magnitude of these associations [27, 66], particularly educational attainment, which may shape occupation, income and overall health. Previous studies investigating cognitive differences between men and women have varied in the identification of the most relevant contributing factors. For example, one study reported that higher waist circumference and mental health symptoms were more strongly associated with cognitive decline and dementia risk in women [67], whereas another study of adults with type 2 diabetes found that traditional risk factors did not explain differences in cognitive impairment prevalence [68]. Although these cohorts differ from the Whitehall II sample, the findings highlight the need to identify the extent to which modifiable risk factors contribute to differences in cognitive ageing as many risk factors vary by sex [26, 69]. This may be particularly relevant given that dementia frequently reflects coexisting AD, vascular, Lewy body and other pathologies. Future studies should also consider neuroimaging and biomarkers of cerebrovascular and neurodegenerative pathology to further elucidate the mechanisms underlying sex differences in cognitive ageing.

Despite observing sex differences in cognitive performance and decline, sex was not significantly associated with dementia risk over 18 years of follow‐up in both crude and adjusted models. A possible explanation for this is the relatively small number of dementia cases in the sample (*n* = 289). This is supported by a meta‐analysis of 29,850 participants (58% women) from 21 cohorts [67]. The pooled estimate showed a greater risk of dementia in women, compared to men, but when comparing at country level, many cohorts did not show sex differences in dementia risk, due to insufficient statistical power. Another explanation could be that in the Whitehall II cohort of civil servants, risk factors were more likely to be similar between the sexes. One study which included 429,340 individuals (50% women) matched by age at baseline found that men had a higher incidence of dementia, but this was largely mediated by health‐related conditions and specific blood and urine biomarkers including APOE, creatinine, low‐density lipoprotein cholesterol (LDL‐C) and lymphocyte percentage [70].

The present findings should also be considered in light of the multifactorial nature of dementia, as different underlying pathologies may contribute to dementia risk. As biomarker data were not included in the present analysis, we could not determine whether the lack of a sex difference in dementia incidence reflected similarities or differences in the presence or extent of underlying pathology. Furthermore, the absence of a sex difference in dementia risk despite poorer performance and decline in fluency among men suggests that differences in specific cognitive domains may not necessarily translate into differences in dementia risk. Future research could therefore consider broader socioecological and life‐course factors, including gender inequality, healthy life expectance and inequality in life expectancy, male secondary education and male survival probability [71]. A socioecological perspective on longevity may provide a useful framework for examining these factors alongside biological processes, providing context for understanding why sex differences in midlife cognition may not translate to differences in dementia risk in later life.

Sex differences in cognitive performance were assessed in 4826 participants across two birth cohorts defined by major historical events: those born in 1935–44 (World War II cohort) and 1945–54 (Post‐World War II cohort). Individuals in the older cohort were born during a period of substantial social and economic disruption, whereas the later cohort experienced post‐war recovery, including expanded access to education and healthcare.

After applying Bonferroni correction (*p* ≤ 0.005), no three‐way interactions between sex, time and birth cohort reached statistical significance. There was, however, a nominal indication of cohort‐specific variation in semantic fluency trajectories. Overall, patterns suggested steeper decline in semantic fluency among men in the older cohort, with weaker or no clear sex differences in the later cohort and other cognitive domains, indicating a possible attenuation of sex differences across cohorts.

These patterns are broadly consistent with previous cohort studies reporting higher cognitive performance in more recent birth cohorts, potentially reflecting improvements in cognitive reserve and population health [72]. However, evidence on sex‐specific cognitive ageing trajectories is mixed, with some studies reporting differing or even opposing patterns across cohorts [73]. Discrepancies may relate to differences in cohort composition, age range at follow‐up and the age at which sex differences in decline emerge.

These cohort differences may reflect long‐term educational and occupational changes across the 20^th^ century. Despite reforms such as the 1944 Education Act, gender disparities in education and occupation likely persisted and may have influenced cognitive reserve and later‐life cognitive trajectories [74, 75]. Importantly, cohort effects were observed without differences in dementia incidence, suggesting that these findings reflect variation in cognitive ageing trajectories rather than disease risk.

APOE‐Ꜫ4 carrier status did not robustly modify the association between sex and cognitive performance after correction for multiple testing. However, there was a nominally significant trend suggesting that men who were homozygotes (Ꜫ4/Ꜫ4) showed faster decline in phonemic fluency, and male heterozygotes showed faster decline in semantic fluency compared to women who were heterozygotes over 16 years of follow‐up. This pattern aligns with some previous observational studies that have shown an effect of APOE‐Ꜫ4 on cognition and rates of cognitive decline [76–79]. It is also broadly consistent with some evidence suggesting that male APOE‐Ꜫ4 carriers have a greater risk of decline [76], although not with other studies reporting greater decline among female Ꜫ4 carriers [80, 81].

Differences in findings may relate to sample characteristics as the Whitehall cohort is based on a sample of healthy individuals, while the previous studies included individuals with MCI and dementia. One study also showed that decline in memory was faster in female APOE‐Ꜫ4 heterozygous carriers [82]. This study had a smaller sample size (*n* = 644) but a longer follow‐up period of 27 years. Similarly, we found that female carriers of the APOE‐Ꜫ4 allele did experience faster decline in memory compared to male APOE‐Ꜫ4 carrier, but this difference did not reach statistical significance. An explanation could be that in the current study, men and women had a mean age of 55.6 years and were followed up for over 16 years. A systematic review of 58,000 adults aged 55–85 years similarly found that APOE‐Ꜫ4 impacted women’s memory performance in middle age but not in later life [23].

### 4.1. Strengths and Limitations

Major strengths of the study are its large sample size, repeated measures of cognitive assessments, comprehensive ascertainment of dementia status, a follow‐up period of over 16 years and data collection on many covariates. The study also allowed for the analysis of birth year cohort, which is important when assessing sex differences as social gradients have evolved over time in the UK. Another key strength is the inclusion of verbal memory and verbal fluency measures, which are sensitive markers of early cognitive decline and cognitive ageing. Reduced performance in these domains has been associated with earlier cognitive deterioration, including subtle deficits in MCI populations [38, 83].

It is important to note several limitations in this study. First, the study population consisted of mostly men (71.2%) and participants of white ethnic background (91.4%). Second, the study recruited British civil servants, and therefore findings may not be fully generalisable to the wider population due to their healthier and more socioeconomically advantaged nature of the cohort. Third, a limitation of measuring only memory and fluency is that the cognitive battery did not cover all cognitive domains, for example, visuospatial, delayed memory and attention. A more comprehensive battery would more accurately measure cognitive impairment. Fourth, the Whitehall II cohort had limited cognitive data available for participants in the oldest‐old age range, since baseline cognitive assessment began at Phase 5 (mean age: 55.5 years) with follow‐up over 16 years, meaning few participants reached the oldest‐old age (80) during the study period, limiting the opportunity to explore sex differences in cognition at advanced aged. Fifth, although some interaction effects remained significant after correction for multiple testing, others did not, suggesting the study may have been underpowered to detect smaller effect modifications, representing a limitation. Sixth, there was an absence of AD and other neurodegenerative biomarkers in the present analysis. Seventh, although participants were free from clinically diagnosed dementia at baseline, biomarker data would have allowed more direct characterisation of underlying pathological processes and their relationship with cognitive trajectories. In the absence of such information, we cannot determine whether the observed domain‐specific differences in fluency reflect differences in AD pathology, cerebrovascular disease, other neurodegenerative processes or mixed pathology. Similarly, the absence of observed differences in memory and dementia does not necessarily indicate an absence of underlying pathological differences, as these may not have been detectable through the outcomes examined. Finally, despite including education, occupation and marital status as SES indicators, annual household income may still be a misleading measure, since in this sample, women were more likely to live alone, which could inflate their apparent household income relative to men in multiperson household.

## 5. Concluding Remarks

Sex differences in cognitive outcomes have often been examined. Sex differences in cognitive performance and decline after adjusting for covariates were observed. Compared to women, men experienced faster rates of cognitive decline in phonemic and semantic fluency. These results suggest that the female advantage in cognition may reflect (i) greater cognitive resilience in women, (ii) other unmeasured or residual covariates not accounted for in this analysis and (iii) biological or life‐course factors, which were not examined in this analysis. Results from the birth cohort analysis suggest that sex differences in covariates such as educational attainment and occupation were smaller in later birth cohorts. This may be beneficial, as sex differences in the rate of decline were not observed in memory or phonemic fluency. The only instance where the rate of decline differed by sex was in the older 1935–44 birth cohort for semantic fluency, where women experienced a slower rate of decline than men. This may warrant further investigation.

Many studies have investigated the association between sex and cognition. However, to date, we have yet to fully understand the drivers behind observed sex differences. Future studies need to unravel the factors contributing to this difference and should assess the interaction between biological and nonbiological factors as it is likely that this difference is driven by both components given that to date, we have not been able to conclusively identify the risk factors driving sex differences. Research should also consider the temporal dynamics of cognitive decline preceding dementia diagnosis. Rather than assuming a constant rate of decline, longitudinal studies could examine whether cognitive trajectories change in the years preceding clinical diagnosis and whether the timing or magnitude of change varies by sex and age at onset [84]. Furthermore, longitudinal biomarker assessments may help determine whether sex differences in cognitive trajectories correspond to differences in the timing or accumulation of specific pathological processes and their co‐occurrence. Finally, rapid advances in artificial intelligence (AI) and Internet of Things (IoT) [85] may facilitate more frequent longitudinal monitoring of cognitive trajectories, helping to clarify timing and interaction of these factors in the period preceding dementia and whether these patterns differ between the sexes.

## Author Contributions

Sima Toopchiani: conceptualisation, formal analysis, methodology, validation, writing–original draft and writing–review and editing, Shireen Sindi: conceptualisation, methodology, supervision and writing–review and editing, Leila Janani: methodology, writing–review and editing and Oliver Robinson: conceptualisation, methodology, supervision and writing–review and editing.

## Funding

No funding was received for this research.

## Conflicts of Interest

The authors declare no conflicts of interest.

## Supporting Information

Additional supporting information can be found online in the Supporting Information section.

## Supporting information


**Supporting Information** Table S1: Number and percentage of missing values for key variables used in the Whitehall II cohort. Table S2: Multiple regression analyses with progressive adjustment for confounders (Models 1–3) assessing the association between sex and baseline cognitive performance in memory and fluency (complete‐case analysis, *N* = 3964). Table S3: Mixed model analyses assessing the interaction between sex and time on the annual mean change in memory and fluency, with progressive adjustment for baseline covariates (*N* = 5047). Table S4: Mixed‐effects model analyses assessing the interaction between sex and time on the annual change in memory and fluency, adjusted for baseline covariates (complete‐case analysis, *N* = 3624). Table S5: Birth cohort–stratified mixed‐effects model analyses assessing the interaction between sex and time on annual change in memory and fluency in the 1935–44 versus 1945–54 birth cohorts (imputed analysis, *N* = 4826). Figure S1: Annual change in phonemic fluency by sex and APOE‐Ꜫ4 carrier status (noncarriers, heterozygotes and homozygotes). Error bars represent 95% confidence intervals. Models were adjusted for baseline age. Table S6: APOE‐stratified mixed‐effects model analyses assessing the interaction between sex and time on annual change in verbal memory and fluency among noncarriers, heterozygotes and homozygotes (imputed analyses, *N* = 4455).

## Data Availability

The dataset supporting the conclusions of this article is available in the Dementias Platform UK (DPUK) repository, DPUK Data Portal, subject to approval through the DPUK data access process and the governance requirements of the Whitehall II study.
