# Supplementary material for: Sex Differences in Domain‐Specific Cognitive Trajectories and Dementia Risk: Evidence From the Whitehall II Cohort
Source: J Aging Res. 2026 Oct 28;2026:1953393. doi: 10.1155/jare/1953393 (PMC13717236; doi:10.1155/jare/1953393)
Supplement: Supplementary file 1 [file JARE-2026-1953393-s001.docx]

**Supplementary Materials**

**Table S1:** Number and percentage of missing key variables used in the Whitehall II cohort

| Variable | N | n=missing | % missing |
| --- | --- | --- | --- |
| ID | 10,308 |  |  |
| Died | 306 |  |  |
| Withdrawn | 752 |  |  |
| Age | 7870 | 2438 | 23.7% |
| Memory | 5995 | 4313 | 41.8 |
| Fluency (words) | 6007 | 4301 | 41.7 |
| Fluency (animals) | 6014 | 4294 | 41.7 |
| Date of participation YYMM | **5920** | 0 | 0.0 |
| Demographics | | | |
| Year of birth | 5920 | 0 | 0.0 |
| Sex | 5920 | 0 | 0.0 |
| Ethnicity | 5919 | <5 | 0.1 |
| Socioeconomic | | | |
| Highest ever qualification | 5812 | 108 | 1.8 |
| Occupation | 5868 | 52 | 0.9 |
| Household income | 4956 | 964 | 16.3 |
| Married/Cohabiting | 5729 | 191 | 3.2 |
| Cardiovascular | | | |
| BMI | 5193 | 727 | 12.3 |
| Systolic BP | 5902 | 18 | 0.3 |
| Diastolic BP | 5902 | 18 | 0.3 |
| Drug class: Antihypertensive | 5874 | 46 | 0.8 |
| On diabetes medication | 5874 | 46 | 0.8 |
| Fasting glucose classification | 5842 | 78 | 1.3 |
| Diabetes diagnosed | 5915 | 5 | 0.1 |
| Stroke | 5920 | 0 | 0.0 |
| CVD | 5874 | 46 | 0.8 |
| Lifestyle | | | |
| Alcohol units (per week) | 5739 | 181 | 3.1 |
| Smoking status | 5838 | 82 | 1.4 |
| Frequency of fruit & vegetables (monthly) | 5739 | 181 | 3.1 |
| Sleep (hours per night) | 5749 | 171 | 2.9 |
| WHO physical activity | 5776 | 144 | 2.4 |
| Other Chronic Conditions | | | |
| Cancer | 5789 | 131 | 2.2 |
| Depression | 5703 | 217 | 3.7 |
| Anxiety subscale | 5717 | 203 | 3.4 |
| APOE (number of Ꜫ4 alleles) | 4495 | 1425 | 24.1 |

**Table S2:** Multiple regression analyses with progressive adjustment for confounders (models 1-3) assessing the association between sex and cognitive performance in memory and fluency at baseline (complete-case analysis, *N*=3,964)

| **Model No.** | **Difference in mean scores (β) (95%CI)** | **P** |
| --- | --- | --- |
| **Cognitive Domain** | **Memory** | |
| **Reference: Women** | - | - |
| **Model 1** | -0.09 (0.1, -0.03) | 0.001 |
| **Model 2** | -0.2 (-0.3, -0.2) | <0.0001 |
| **Model 3** | -0.2 (-0.3, -0.02) | <0.0001 |
|  | **Phonemic Fluency** | |
| **Reference: Women** | - | - |
| **Model 1** | -0.06 (-0.1, -0.008) | 0.05 |
| **Model 2** | -0.08 (-0.2, -0.02) | 0.03 |
| **Model 3** | -0.08 (-0.2, 0.002) | 0.06 |
|  | **Semantic Fluency** | |
| **Reference: Women** | - | - |
| **Model 1** | 0.1 (0.05, 0.2) | <0.0001 |
| **Model 2** | -0.2 (-0.3, -0.2) | <0.0001 |
| **Model 3** | -0.2 (-0.3, -0.1) | <0.0001 |

Model 1: age & ethnicity, Model 1 + 2: education, household annual income, occupation & marital status,

Model 1, 2 + 3: BMI, hypertension, diabetes, stroke, CVD, alcohol consumption, smoking status, diet, sleep, physical activity, cancer, depression & anxiety. Notes: Nominal significance threshold: p <0.05 and Bonferroni-corrected significance threshold: p <0.005.

**Table S3**: Mixed model analyses assessing the interaction between sex and time on the annual mean change in memory and fluency, with progressive adjustment for baseline covariates (*N= 5,047*).

| **Interaction between male & time** | Change in mean scores per year **(β) (95% CI)** | **P** |
| --- | --- | --- |
| **Memory** | | |
| **Ref: Women** | - | - |
| **Model 1** | 0.3 (-1.4, 2.1) | 0.7 |
| **Model 2** | 0.3 (-1.5, 2.1) | 0.7 |
| **Model 3** | 0.3 (-1.5, (2.1) | 0.7 |
| **Model 4** | 0.3 (-1.4, 2.1) | 0.7 |
| **Model 5** | 0.3 (-1.4, 2.1) | 0.7 |
| **Phonemic Fluency** | | |
| **Ref: Women** | - | - |
| **Model 1** | -1.7 (-2.9, -0.4) | 0.008 |
| **Model 2** | -1.7 (-2.9, -0.5) | 0.007 |
| **Model 3** | -1.7 (-2.9, -0.5) | 0.007 |
| **Model 4** | -1.7 (-2.9, -0.5) | 0.007 |
| **Model 5** | -1.7 (-2.9, -0.5) | 0.007 |
| **Semantic Fluency** | | |
| **Ref: Women** | - | - |
| **Model 1** | -2.6 (-4.1, -1.2) | <0.0001 |
| **Model 2** | -2.7 (-4.1, -1.2) | <0.0001 |
| **Model 3** | -2.6 (-4.1, -1.2) | <0.0001 |
| **Model 4** | -2.6 (-4.1, -1.2) | <0.0001 |
| **Model 5** | -2.6 (-4.1, -1.2) | <0.0001 |

Model 1: age & ethnicity, Model 1 + 2: education, household annual income, occupation & marital status, Model 1, 2 + 3: BMI, hypertension, diabetes, stroke, & CVD, Model 1, 2, 3 + 4: alcohol consumption, smoking status, diet, sleep & physical activity, Model 1, 2, 3, 4 + 5: cancer, depression & anxiety. Notes: Nominal significance threshold: p <0.05 and Bonferroni-corrected significance threshold: p <0.005.

**Table S4**: Mixed model analyses assessing the interaction between sex and time on the annual mean change in memory and fluency, adjusted for baseline covariates (complete-case analysis, *N*=3,624)

| **Fully adjusted Model** | Change in mean scores per year **(β) (95% CI)** | **P** | Change in mean scores **5** years **(β) (95% CI)** | **P** |
| --- | --- | --- | --- | --- |
| **Memory** | | | | |
| **Ref: Women** | - | - | - | - |
| **1. Main effect: Male^1^** | -0.2 (-0.3, -0.1) | <0.0001 | -0.2 (-0.3, -0.1) | <0.0001 |
| **2. Interaction between male & time^2^** | 0.2 (-1.9, 2.3) | 0.9 | 0.9 (-9.7, 11.6) | 0.9 |
| **Phonemic Fluency** | | | | |
| **Ref: Women** | - | - | - | - |
| **1. Main effect: Male^1^** | -0.1 (-0.2, -0.09) | <0.0001 | -0.1 (-0.2, -0.09) | <0.0001 |
| **2. Interaction between male & time^2^** | -1.8 (-3.3, -0.4) | 0.01 | -9.2 (-16.5, -1.9) | 0.01 |
| **Semantic Fluency** | | | | |
| **Ref: Women** | - | - | - | - |
| **1. Main effect: Male^1^** | -0.1 (-0.2, -0.05) | 0.001 | -0.1 (-0.2, -0.05) | 0.001 |
| **2. Interaction between male & time^2^** | -2.9 (-4.6, -1.2) | 0.001 | -14.3 (-22.8, -5.8) | 0.001 |

Adjusted for baseline age, ethnicity, education, occupation, household annual income, marital status, BMI, CVD, hypertension, diabetes, stroke, alcohol consumption, diet, physical activity, cancer, depression, and anxiety. ^1^Difference in mean scores, men compared to women. ^2^Change in mean scores per year in men compared to women. Notes: Nominal significance threshold: p <0.05 and Bonferroni-corrected significance threshold: p <0.005.

**Table S5:** Birth-cohort mixed-model regression analyses assessing the interaction between sex and time on mean annual change in memory and fluency in the 1935-44 versus 1945-54 birth year cohort (imputed model, *N*= 4,826)

| **Model No.** | **N** | **Change in mean scores per year (ß) (95% CI)** | **P** |
| --- | --- | --- | --- |
|  |  | **Memory** | |
| Ref: Women |  | - | - |
| 1. Two-way interaction between male and time (1935-44) | 2545 | 0.6 (-1.7, 3.0) | 0.6 |
| 2. Two-way interaction between male and time (1945-55) | 2281 | 0.2 (-1.3, 1.8) | 0.8 |
| 3. Three-way interaction between male, time & 1935-44 birth year | 2545 | 1.0 (-1.7, 3.6) | 0.5 |
| 4. Three-way interaction between male, time & 1945-54 birth year | 2281 | 0.1 (-2.5, 2.8) | 0.9 |
|  |  | **Phonemic Fluency** | |
| Ref: Women |  | - | - |
| 1. Two-way interaction between male and time (1935-44) | 2545 | -1.8 (-3.5, -0.1) | 0.04* |
| 2. Two-way interaction between male and time (1945-55) | 2281 | -1.5 (-2.6, -0.4) | 0.007* |
| 3. Three-way interaction between male, time & 1935-44 birth year | 2545 | -1.5 (-3.3, 0.3) | 0.1 |
| 4. Three-way interaction between male, time & 1945-54 birth year | 2281 | -1.4 (-3.2, 0.4) | 0.1 |
|  |  | **Semantic Fluency** | |
| Ref: Women |  | - | - |
| 1. Two-way interaction between male and time (1935-44) | 2545 | -3.1 (-5.0, -1.2) | 0.002† |
| 2. Two-way interaction between male and time (1945-55) | 2281 | -2.3 (-3.6, -0.1) | 0.001† |
| 3. Three-way interaction between male, time & 1935-44 birth year | 2545 | -2.8 (-4.9, -0.7) | 0.01* |
| 4. Three-way interaction between male, time & 1945-54 birth year | 2281 | -1.8 (-3.9, 0.3) | 0.09 |

Adjusted for baseline age, ethnicity, education, occupation, household annual income, marital status, BMI, CVD, hypertension, diabetes, stroke, alcohol consumption, diet, physical activity, cancer, depression, and anxiety. Notes: Nominal significance threshold: p <0.05 (*). Bonferroni-corrected significance threshold: p <0.005 (†). Values meeting the Bonferroni-corrected threshold are marked †; those meeting only the nominal threshold are marked *.

*p=0.05

**Figure S1:** Change in mean difference in phonemic fluency per year stratified by sex and APOE-Ꜫ4 carrier status; ‘non-carrier’ versus ‘Ꜫ4-carrier (heterozygote)’ versus ‘Ꜫ4/Ꜫ4 carrier (homozygote)’. Mean estimate (Est) and error bars showing 95% confidence intervals. Model adjusted for baseline age.

**Table S6:** APOE-stratified mixed-model regression analyses assessing the interaction between sex and time on annual change in verbal memory and fluency in non-carriers, heterozygotes, and homozygotes (imputed analyses, *N*= 4,455)

| **Model No.** | **N** | Change in mean scores per year **(β) (95% CI)** | **P** |
| --- | --- | --- | --- |
| **Memory** | | **Age adjusted Model** | |
| **Ref: Women** |  |  |  |
| **1.Interaction between male and time (non-carrier)** | **3217** | 0.8 (-1.3, 3.0) | 0.5 |
| **2.Interaction between male and time**  **(Ꜫ4-hetrozygote)** | **1133** | -1.1 (-5.1, 2.8) | 0.6 |
| **3.Interaction between sex and time**  **(Ꜫ4-homozygote)** | **105** | 2.3 (-12.3, 17.0) | 0.8 |
| **4. 3-way interaction (male*time*Ꜫ4- heterozygote)** | **1133** | 2.0 (-2.9, 6.8) | 0.4 |
| **5. 3-way interaction (male*time*Ꜫ4-homozygote)** | **105** | -2.7 (-17.8, 12.4) | 0.7 |
| **Phonemic Fluency** | | **Age adjusted Model** | |
| **Ref: Women** | **-** | - | - |
| **1.Interaction between male and time (non-carrier)** | **3217** | -2.0 (-3.5, -0.5) | 0.008* |
| **2.Interaction between male and time**  **(Ꜫ4-hetrozygote)** | **1133** | 0.3 (-2.5, 3.0) | 0.8 |
| **3.Interaction between sex and time**  **(Ꜫ4-homozygote)** | **105** | -10.9 (-20.8, -1.0) | 0.04* |
| **4. 3-way interaction (male*time*Ꜫ4- heterozygote)** | **1133** | 3.3 (0.05, 6.6) | 0.05* |
| **5. 3-way interaction (male*time*Ꜫ4-homozygote)** | **105** | -2.0 (-12.0, 7.9) | 0.7 |
| **Semantic Fluency** | | **Age adjusted Model** | |
| **Ref: Women** | **-** | - | - |
| **1.Interaction between male and time (non-carrier)** | **3217** | -2.0 (-3.8, -0.2) | 0.03* |
| **2.Interaction between male and time**  **(Ꜫ4-hetrozygote)** | **1133** | -3.5 (-6.8, -0.2) | 0.04* |
| **3.Interaction between sex and time**  **(Ꜫ4-homozygote)** | **105** | -4.4 (-16.2, 7.3) | 0.5 |
| **4. 3-way interaction (male*time*Ꜫ4- heterozygote)** | **1133** | -1.8 (-5.8, 2.2) | 0.4 |
| **5. 3-way interaction (male*time*Ꜫ4-homozygote)** | **105** | 11.5 (-0.6, 23.7) | 0.06 |

Notes: Nominal significance threshold: p <0.05 (*). Bonferroni-corrected significance threshold: p <0.004 (†). Values meeting the Bonferroni-corrected threshold are marked †; those meeting only the nominal threshold are marked *.
